# ID 127 - ESMO-MCBS e Conitec: o valor de medicamentos oncológicos para médicos corresponde ao valor percebido pelos gestores e pagadores no Sistema Único de Saúde?

**DOI:** 10.5327/2237-9622.2025.v34s1.127

**Published:** 2025-11-25

**Authors:** Ludmila Peres Gargano, Mariana Millan Fachi, Vinicius Lins Ferreira, Layssa Andrade Oliveira, Haliton Alves de Oliveira, Rosa Camila Lucchetta

**Keywords:** valor terapêutico, oncologia, Avaliação de Tecnologias em Saúde, tomada de decisão em saúde

## Abstract

**Introdução:**

O conceito de "valor" de uma tecnologia em saúde pode ser ambíguo e é intrinsecamente dependente da perspectiva em questão. Todavia, é inquestionável que a dimensão do benefício clínico produzido quando comparado às alternativas disponíveis pode representar grande parte deste valor. Nesse sentido, a Sociedade Europeia de Oncologia Médica (ESMO) desenvolveu a ESMO-MCBS (do inglês, ), uma ferramenta validada e reprodutível para padronizar a classificação do benefício clínico relativo de terapias oncológicas, considerando a eficácia e a segurança. Sem embargo, outros aspectos são igualmente relevantes sob a perspectiva da tomada de decisão sobre reembolso e incorporação como aqueles adotados pela Conitec como a custo-efetividade e o impacto orçamentário. Assim, este trabalho se propõe a discutir o papel e a relevância da ferramenta ESMO-MCBS na tomada de decisões em saúde sob a perspectiva do Sistema Único de Saúde, ao avaliar recomendações da Conitec sobre terapias oncológicas e as respectivas classificações recebidas na ferramenta.

**Método:**

Trata-se de uma análise documental, na qual foi selecionada uma amostra por conveniência de Relatórios de Recomendação submetidos para avaliação da Conitec, de 2022 a 2024, sobre terapias para tumores sólidos. Dos relatórios selecionados, foram extraídas informações sobre a demanda (tecnologia, indicação e comparador) e os principais resultados relacionados aos benefícios clínicos (eficácia e segurança) e econômicos (custo anual de tratamento, razão de custo efetividade incremental [RCEI] e impacto orçamentário [IO] acumulado em cinco anos). Paralelamente, foram realizadas buscas no site da ESMO-MCBS para identificar os escores pela ferramenta para cada tecnologia selecionada na amostra, considerando a mesma indicação e comparador. Para tecnologias no contexto não curativo a ferramenta classifica o benefício de 1 (pontuação mínima) a 5 (pontuação máxima), e quanto maior a nota, maior o benefício clínico produzido.

**Resultados:**

Foram avaliadas oito terapias oncológicas, das quais a maioria (n=6) recebeu recomendação final desfavorável para incorporação pela Conitec, sendo que para estas, as notas ESMO-MCBS foram 3 (n=1), 4 (n=4) e 5 (n=1). O nivolumabe para câncer de cabeça e pescoço foi o único com nota máxima (ESMO-MCBS 5/5) e teve o maior custo anual de tratamento (R$ 401.642). Duas tecnologias receberam recomendação final favorável: abiraterona para câncer de próstata e durvalumabe para câncer de pulmão avançado (nota ESMO-MCBS 2 e 4, respectivamente) sendo as únicas com RCEI dentro do limiar de custo-efetividade do SUS (R$ 36.759 e R$ 118.192 por QALY, respectivamente). Apesar de apresentar menor nota na ferramenta, a abiraterona para câncer de próstata teve a menor RCEI e custo anual (R$ 8.468) de tratamento entre as tecnologias avaliadas.

**Conclusão:**

Embora as abordagens da ESMO e Conitec convirjam na importância ao privilegiar tecnologias que produzam maiores benefícios clínicos à sociedade, os aspectos econômicos parecem exercer um peso maior nas decisões da Conitec, o que está alinhado com os preceitos de sustentabilidade do sistema. Diferentemente de outros estudos que sugeriram correlação entre a classificação e a direção da recomendação de outras agências (e.g. NICE), a nota da ESMO-MCBS parece não ser fator determinante para a decisão da Conitec.

